# Optimized Clustering Algorithm for Comparative Analysis of Different Prenatal Corticosteroid Neurological Deficits in Premature Infants through Magnetic Reasoning Imaging (MRI)

**DOI:** 10.1155/2021/6179177

**Published:** 2021-07-26

**Authors:** Shu Zhao

**Affiliations:** Department of Paediatrics, Anshan Central Hospital, Anshan 114001, Liaoning, China

## Abstract

**Objective:**

This study aimed to explore the application of different prenatal corticosteroids in the assessment of neurological deficits and prognosis in premature infants through Magnetic Reasoning Imaging (MRI) under optimized cluster algorithm.

**Methods:**

100 pregnant women with threatened preterm labor were retrospectively analyzed, in which 38 pregnant women with lasting threatened preterm labor (group A) were treated with multiple courses of antenatal corticosteroids (dexamethasone treatment) and 62 cases of pregnant women with threatened preterm labor (group B) were treated with single course of dexamethasone treatment. Craniocerebral MRI images based on optimal clustering algorithm were used to examine neonates. Neonatal hypoxic-ischemic encephalopathy (HIE) rate, serum neuron-specific enolase (NSE) concentration, neonatal behavioral neurological score (NBNA), respiratory distress syndrome (RDS) rate, perinatal mortality, neonatal birth weight, and maternal complications rate of two groups were compared.

**Results:**

Compared with other traditional image segmentation algorithms, this algorithm had the best segmentation effect, the shortest running time (1.43 s), the least number of iterations (5 times), and the highest segmentation accuracy (97.98%). There was no significant difference in the HIE rate, serum NSE concentration, NBNA score, RDS score, and perinatal mortality in group A and group B (*P* > 0.05). Compared with group B, neonates' body weight in group A was decreased, while the maternal complication rate in group A was increased (*P* < 0.05).

**Conclusion:**

MRI images based on optimized clustering algorithm can be used in the diagnosis of neonatal hypoxic-ischemic encephalopathy. There is no significant difference in the application of different antenatal corticosteroids affecting premature nerve function defect and prognosis, but multiple courses of antenatal corticosteroids can affect neonatal body mass and increased maternal complications to a certain extent; therefore, before threatened premature delivery treatment, the pros and cons of multiple courses of antenatal corticosteroids should fully be considered and in the treatment, measures should be actively taken to alleviate the side effect.

## 1. Introduction

Premature delivery is a common clinical phenomenon with relatively high clinical incidence. The occurrence of complications such as neonatal acute respiratory distress syndrome, cerebral hypoxia, and others is frequent, and some of the infants may have poor prognosis such as death, which seriously threatens the health and life safety of the newborn and affects the quality of the new generation [1, 2]. Currently, MRI is the preferred method for assessing brain development [3]. MRI images do not have radiation damage and not only can observe the pathological changes of brain damage but also reflect the early metabolic level to a certain extent. It is widely used in the research of neonatal ischemic hypoxic encephalopathy and other brain damage diseases. Medical image segmentation is a key link in the image processing process. After image is processed using clustering algorithms, image segmentation accuracy and detection accuracy are further optimized and improved [4].

It can help the newborn's health and improve prognosis conditions when corticosteroids are used for prematures to promote fetal maturity in order to prevent and treat their complications [5, 6]. In addition, studies have shown that corticosteroids in different courses of treatment have different effects on promoting fetal maturation [7]. Therefore, it is of great significance to investigate the neurological deficits and prognosis in preterm infants treated with different antenatal corticosteroids. However, there is still little research on this aspect, which needs to be confirmed by further research. Therefore, in this study, MRI images based on optimal clustering algorithm were used to evaluate neonatal ischemic hypoxic encephalopathy. Then, the effects of a single course and multiple courses of corticosteroid dexamethasone were retrospectively analyzed on the neurological dysfunction of premature infants and the prognosis of them.

## 2. Materials and Methods

### 2.1. General Information

100 pregnant women with threatened preterm labor in hospital from 2016 to 2020 were retrospective analyzed. The inclusion criteria were a contraction stimulation test was performed for 1 hour at the time of consultation, at least one contraction every 10 minutes, no fetal distress, cervical dilation was less than 3 cm, and the membrane was not broken; and the exclusion criteria were pregnant women with other pregnancy complications and contraindications for fetal pregnancy treatment. All the pregnant women met the inclusion criteria, and those who were not pregnant were excluded. Finally, 100 cases of pregnant women of threatened premature labor were chosen, in which 38 cases of pregnant women with continuous threatened premature labor (group A) and 62 cases of pregnant women with threatened premature labor (group B) were included. This test met ethical standards and was approved by the ethics committee of hospital, and all the patients signed the informed consent forms. There was no statistically significant difference in baseline data between the two groups (*P* > 0.05), which was comparable, as shown in Table 1.

### 2.2. Therapeutic Methods

Group A was treated with multiple courses of prenatal dexamethasone, while group B was treated with a single course of dexamethasone. Both groups received intramuscular injection of dexamethasone 12 mg once a day and got it once again after 24 hours. Within 7 days thereafter, no dexamethasone treatment was given. The result showed that the fetus was still not delivered 7 days after the first intramuscular injection of dexamethasone in group A. In group B, the fetus was delivered within 7 days after the first intramuscular injection of dexamethasone.

### 2.3. MRI Examination

1.5 T high-field magnetic resonance scanning equipment was used to examine all newborns. The examination was started when the patient was in a quiet and peaceful state. If the patient cannot keep quiet, 10% chloral hydrate can be taken 20 minutes before the scan. Then, the examination was started after the patient fell asleep. Scanning was performed in T1W1, T2W1, and FLAIR (fluid attenuated inversion recovery) axis, respectively. The SE sequence was adopted for TIWI, TR was 200 ms, and TE (time of echo) was 4.76 ms; the TSE (turbo spin echo) sequence was used for T2W1, TR was 5570 ms, and TE was 117 ms. The scanning matrix was 256 × 256, the thickness of the scanning layer was 4 mm, and the FOV was 180 mm.

### 2.4. MRI Image Based on Optimized Clustering Algorithm

Due to the complexity of medical MRI images, there is often degradation in the process of semisupervised fuzzy cluster analysis. In the study, an optimized clustering algorithm was proposed for the difficulties encountered in MRI image segmentation. This algorithm was improved based on the semisupervised FCM algorithm [8], and the specific process was as follows.

First, the data and parameters were initialized. Assuming that the number of clusters was *c* in the sample set, the distance *D* was calculated from the cluster center to the sample point.

(1) $$\begin{array}{c} D=\left(\sum_{k=1}^{D}{\left(x_{i,k}-x_{j,k}\right)}^{1/2}\right)={\left\|x_{i}-x_{j}\right\|}_{2.}. \end{array}$$

Iterative update was conducted according to equation (1) to obtain the degree of membership *A*_*ik*_′. Then, the degree of membership matrix *U*=[*A*_*ik*_] was obtained.

(2) $$\begin{array}{c} A_{ik}^{\prime }=\frac{1}{1+\alpha }\left[\frac{1}{\sum_{j=1}^{c}D_{ik}^{2}/D_{jk}^{2}}+\alpha f_{ik}b_{k}\right]. \end{array}$$

After that, the cluster centers were continuously updated according to equation (3), and the set of cluster centers *U*=[*B*_*i*_] was obtained.

(3) $$\begin{array}{c} B_{i}^{\prime }=\frac{\sum_{k=1}^{n}A_{ik}^{2}x_{k}}{\sum_{k=1}^{n}A_{ik}^{2}}. \end{array}$$

If the condition ‖*U*^*t*^ − *U*^(*t* − 1)^‖ < *ε* was met, the iteration stopped. Then, *U* and *V* were output. If the conditions were not met, assuming *b* = *b *+* *1, then it needed to return to equation (1) to restart.

### 2.5. Observation Indicators and Detection Methods

Neonatal hypoxic-ischemic encephalopathy (HIE) rate, serum neuron-specific enolase (NSE) concentration, neonatal behavioral neurological assessment (NBNA), respiratory distress syndrome (RDS) rate, perinatal mortality, neonatal birth weight, and maternal complication rate such as gastrointestinal symptoms, chest distress, increased heart rate, pulmonary edema, retention of urine, and infection of the two groups were compared. At the time of birth and after 3 days, 2 ml of peripheral venous blood was drawn for neonatal serum neuron-specific enolase (NSE) concentration. The blood samples obtained were routinely centrifuged and refrigerated; then, the Eleesys 2010 automatic biochemical analyzer and its testing kits produced by Swiss Roche were used for detecting, in which the testing operation was strictly in accordance with the guidance of the instrument and instructions.

### 2.6. Evaluation Methods

NBNA [9] including behavioral ability, passive muscle tone, active muscle tone, primitive reflex, and general assessments was applied. The score ranges from 0 to 40; unquestionably, the higher the score, the better the behavioral neurodevelopment.

### 2.7. Statistical Methods

SPSS19.0 software was used. The measurement data were calculated as mean ± standard deviation (*x*(−) ± *s*), and the measurement data were expressed as percentage. The chi-square test was used for the comparison of counting data, and the measurement data were in line with the normal distribution. The mean *t*-test of two independent samples was used for the comparison between the measurement data groups, and when *P* < 0.05, it was considered statistically significant.

## 3. Results

### 3.1. Comparison of MRI Image Segmentation Results Based on Optimized Clustering Algorithm

**Figure 1:**
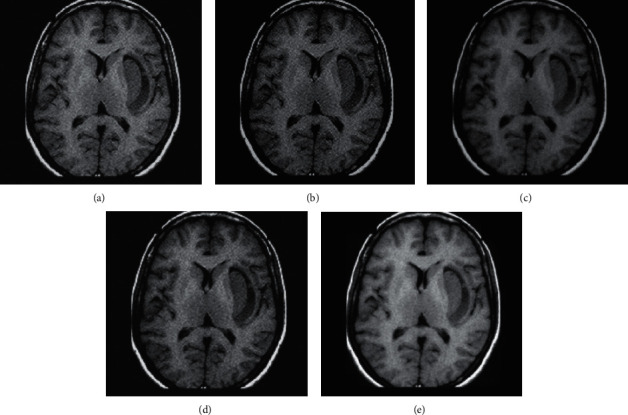
Detection results of different algorithms. (a) The original image; (b) expert segmentation; (c) FCM; (d) semisupervised FCM segmentation; and (e) the algorithm in this study.

In this study, the image segmentation detection algorithm based on the optimized clustering algorithm was used in MRI image processing. Besides, its results were compared with the results of expert segmentation, FCM, and semisupervised FCM segmentation. The results of the simulation test are shown in Figure 1 and Table 2. Compared with other traditional image segmentation algorithms, this algorithm had the best segmentation effect, the shortest running time (1.43), the least number of iterations (5), and the highest segmentation accuracy (97.98).

### 3.2. MRI Images of Some Children

**Figure 2:**
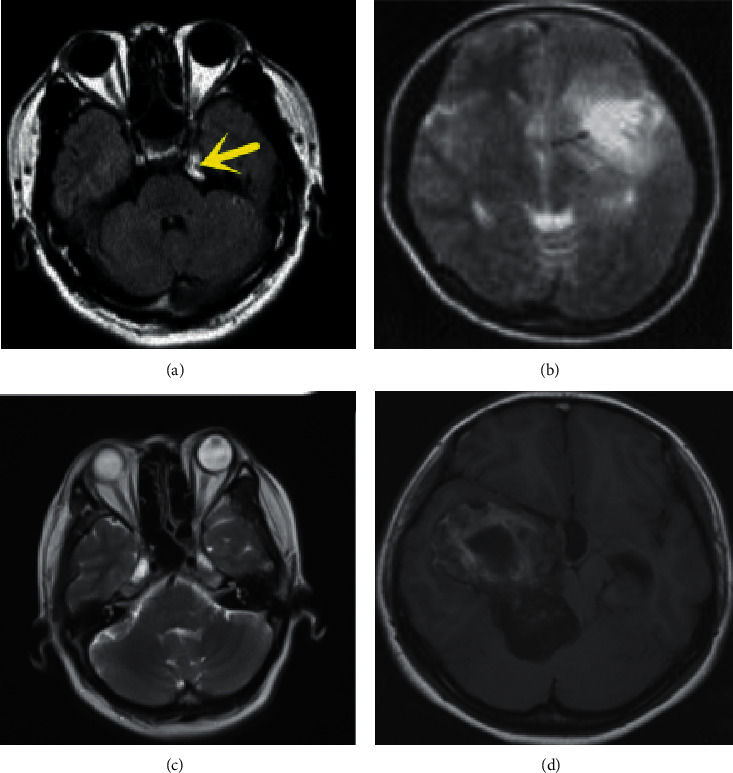
MRI images of (a) a non-HIE child and (b–d) children with HIE.

The main features of MRI images in children with non-HIE were as follows. The yellow matter of the brain presented slightly higher T1W1 and long T2W1 low signal (Figure 2(a)).

Children with HIE had different degrees of brain damage. The MRI images featured cerebral edema, low T1W1 white matter signal, and high T2W1 white matter signal. There were punctate and patchy abnormal signal shadows in brain parenchyma (Figure 2(b)). There was intraventricular and subarachnoid hemorrhage (Figure 2(c)). In addition, subcortical and deep white matter hemorrhage appeared (Figure 2(d)).

### 3.3. Comparison of Neurological Functional Defects in Group A and Group B

There was no significant difference in the HIE rate, serum NSE concentration, NBNA score, RDS score, and perinatal mortality in group A and group B (*P* > 0.05), as shown in Table 3.

### 3.4. Comparison of Neonatal Prognosis between Group A and Group B

There was no significant difference in RDS score and perinatal mortality in group A and group B (*P* > 0.05) (Table 4). Compared with group B, infants' body weight in group A was decreased, and this difference was statistically significant (*P* < 0.05).

### 3.5. Comparison of Maternal Complication Rate between Group A and Group B

Compared with group B, maternal complication rate such as gastrointestinal symptoms, chest distress, increased heart rate, pulmonary edema, retention of urine, and infection in group A were increased and its difference was statistically significant (*P* < 0.05), as shown in Table 5.

## 4. Discussion

The main cause of neonatal perinatal disease and death was premature birth, and the perinatal mortality caused by premature birth accounted for most of the overall perinatal mortality. In recent years, the clinical morbidity of preterm birth has continued to increase and premature infants would have many complications which will further affect the health level of newborns and even survival prognosis [10–12]. In the study, the optimized cluster algorithm was applied to medical MRI image processing. Then, the results were compared with those of expert segmentation, FCM, and semisupervised FCM segmentation. It was found that compared with other traditional image segmentation algorithms, this algorithm had the best segmentation effect and the shortest running time (1.43 s). Besides, the number of iterations was the least (5), and the segmentation accuracy was the highest (97.98). This was consistent with the results of Lu and Yan [13]. Among them, acute respiratory distress syndrome is a common complication. Children with dyspnea are prone to infect hypoxic-ischemic encephalopathy, which leads to hypoxic-ischemic damage of cerebral nerves and triggers dysfunction of the nervous system. Abnormal consciousness, convulsions, and changes in muscle tone in severe cases can lead to the occurrence of adverse prognosis such as disability and even neonatal death [14, 15]. This study also focused on the incidence of complications in preterm infants. The results of this study showed that the incidence of neonatal hypoxic-ischemic encephalopathy was as high as about 28%. It could be told that the neurobehavior was obviously abnormal. Therefore, it was of great significance to take active measures to prevent and cure the complications of neonates to improve their health status and prognosis.

Prenatal use of corticosteroids to promote fetal maturation is an important measure to prevent premature infant complications, and the prevention and treatment of premature infant complications is conducive to the improvement of newborn health and prognosis [16, 17]. However, different treatment courses of corticosteroids have different effects and safety in promoting fetal maturation. Studies have shown that multiple courses of corticosteroids promoting fetal maturation may affect the growth and development of premature fetuses. It can affect the development of the lungs, immune system, internal organs, and nervous system; however, its specific impact is not clear [18]. Therefore, this study analyzed the effects and safety of single- and multiple-course prenatal corticosteroids applied to threatened preterm delivery on neurological deficits and prognosis in preterm infants. The results of this study showed that single- and multiple-course prenatal corticosteroids applied to threatened preterm delivery had little difference in the incidence of hypoxic-ischemic encephalopathy in neonates, the serum NSE concentration at birth and at 3 days of birth, the NBNA score, the incidence of RDS, and the perinatal mortality rate. This was consistent with the results of Roberts et al. (2017) [19] and Crowther et al. (2019) [20]. Moreover, multiple courses of antepartum corticosteroid therapy for preterm labor with persistent premonition can improve the neurological function and prognosis of neonates. Compared with single-course prenatal corticosteroids, multicourse prenatal corticosteroids were used to reduce the weight of neonates with threatened preterm birth, but the incidence rate of complications such as maternal gastrointestinal symptoms, chest distress, increased heart rate, pulmonary edema, retention of urine, and infection were increased. A single course of prenatal corticosteroids to promote fetal maturation is safe and effective. For continuous threatened preterm labor, before giving birth to multicourse corticosteroid treatment, it should be fully considered the possible negative effects on perinatal and pregnant women by weighing the pros and cons, and doctors should fully communicate with pregnant women and their families. When the benefits outweigh the disadvantages and the consents of pregnant women and their families are obtained, multiple courses of prenatal corticosteroid treatment can be carried out. Moreover, at the same time of treatment, perinatal and pregnant women should be closely observed, and active measures should be taken to intervene in abnormal persons in order to minimize the side effects of treatment and improve the prognosis of the mother and child.

## 5. Conclusions

MRI images based on the optimized cluster algorithm can be used in the diagnosis of neonatal HIE. There is no significant difference in the effects of different courses of prenatal corticosteroids on neurological deficits and prognosis in preterm infants. However, when multiple courses of antenatal corticosteroids are used for lasting threatened preterm labor, it can affect the weight of newborns and increase maternal complications. Therefore, before the treatment by antenatal corticosteroids, the pros and cons should be fully weighed, and measures should be taken to alleviate the side effects of treatment, to safely and effectively improve the neurological function and prognosis of premature infants. In addition, the sample size of this study is small, the results of the study may be affected by the large differences in individual samples, and the treatment and testing may be affected by the operator's technique. Therefore, the effects of different prenatal antenatal corticosteroids on neurological deficits and prognosis of premature infants need to be confirmed by a comprehensive study with a large sample size. In summary, the results of this study can provide a reliable basis for the prevention and clinical treatment of neurological deficits in preterm infants.

## Figures and Tables

**Table 1:** Comparison of baseline data between the two groups.

Groups	Number of cases	Age (years)	Production time (times)	Gestational weeks (weeks)	Single birth/twins
Group A	38	26.85 ± 5.88	1.15 ± 1.02	31.25 ± 2.15	36/2
Group B	62	26.71 ± 7.62	1.09 ± 1.01	31.16 ± 2.37	58/4
Statistical magnitude		*t* = 0.103	*t* = 0.287	*t* = 0.191	*χ* ^2^ = 0.059
*P*		0.918	0.775	0.849	0.808

**Table 2:** Performance comparison of different algorithms.

	Running time (s)	Number of iterations (times)	Segmentation accuracy (%)
FCM	5.74	50	91.56
Semisupervised FCM	2145	10500	92.34
Algorithm	1.43	5	97.98

**Table 3:** Comparison of neurological functional defects in group A and group B.

Groups	Number of cases	NBNA score (scores)	NSE at birth (*μ*g/L)	NSE at the 3rd day after birth (*μ*g/L)	HIE (cases (%))
Group A	38	34.45 ± 4.25	26.28 ± 7.21	33.22 ± 9.58	8 (21.05)
Group B	62	33.65 ± 4.72	27.11 ± 7.42	32.16 ± 10.05	15 (24.19)
Statistical magnitude		*t* = 0.854	*t* = 0.549	*t* = 0.521	*χ* ^*2*^ = 0.131
*P*		0.395	0.584	0.604	0.717

**Table 4:** Comparison of neonatal prognosis between group A and group B.

Groups	Number of cases	Body mass of the infants (g)	Perinatal death (cases (%))	RDS (cases (%))
Group A	38	2721.42 ± 241.15	1 (2.63)	3 (7.89)
Group B	62	2511.72 ± 203.36	0 (0.00)	4 (6.45)
Statistical magnitude		*t* = 4.661	*χ* ^2^ = 1.648	*χ* ^2^ = 0.075
*P*		<0.001	0.380	1.000

**Table 5:** Comparison of maternal complication rate between group A and group B.

Groups	Number of cases	Gastrointestinal symptoms (cases)	Chest distress (cases)	Increased heart rate (cases)	Pulmonary edema (cases)	Retention of urine (cases)	Infection (cases)	The overall incidence (cases (%))
Group A	38	1	0	0	0	0	0	1 (2.63)
Group B	62	5	3	2	1	1	1	13 (20.96)
*χ* ^2^								6.579
*P*								0.010

## Data Availability

The data used to support the findings of this study are available from the corresponding author upon request.
